# Quantitative trait locus mapping of *Populus* bark features and stem diameter

**DOI:** 10.1186/s12870-017-1166-4

**Published:** 2017-11-28

**Authors:** Roba Bdeir, Wellington Muchero, Yordan Yordanov, Gerald A. Tuskan, Victor Busov, Oliver Gailing

**Affiliations:** 1 School of Forest Resources and Environmental Science, Michigan Technological University, 1400 Townsend Drive, Houghton, MI 49931 USA; 2 Biosciences Division, Oak Ridge National Laboratory, 1 Bethel Valley Road, Oak Ridge, TN 37831 USA; 3 Departement of Biology, Eastern Illinois University, 600 Lincoln Ave, Charleston, IL 61920 USA; 4 Present address: Forest Genetics and Forest Tree Breeding, Faculty of Forest Sciences, University of Göttingen, Büsgenweg 2, 37077 Göttingen, Germany

**Keywords:** Bark texture, Bark thickness, Diameter, Quantitative trait loci (QTL), Stem, *Populus trichocarpa*

## Abstract

**Background:**

Bark plays important roles in photosynthate transport and storage, along with physical and chemical protection. Bark texture varies extensively among species, from smooth to fissured to deeply furrowed, but its genetic control is unknown. This study sought to determine the main genomic regions associated with natural variation in bark features and stem diameter. Quantitative trait loci (QTL) were mapped using an interspecific pseudo-backcross pedigree (*Populus trichocarpa* x *P. deltoides* and *P. deltoides*) for bark texture, bark thickness and diameter collected across three years, two sites and three biological replicates per site.

**Results:**

QTL specific to bark texture were highly reproducible in shared intervals across sites, years and replicates. Significant positive correlations and co-localization between trait QTL suggest pleiotropic regulators or closely linked genes. A list of candidate genes with related putative function, location close to QTL maxima and with the highest expression level in the phloem, xylem and cambium was identified.

**Conclusion:**

Candidate genes for bark texture included an ortholog of *Arabidopsis* ANAC104 (PopNAC128), which plays a role in lignified fiber cell and ray development, as well as Pinin and Fasciclin (PopFLA) genes with a role in cell adhesion, cell shape and migration. The results presented in this study provide a basis for future genomic characterization of genes found within the QTL for bark texture, bark thickness and diameter in order to better understand stem and bark development in *Populus* and other woody perennial plants. The QTL mapping approach identified a list of prime candidate genes for further validation using functional genomics or forward genetics approaches.

**Electronic supplementary material:**

The online version of this article (10.1186/s12870-017-1166-4) contains supplementary material, which is available to authorized users.

## Background

Bark, the outermost surface of stems and branches in woody plants, encompasses all tissues outside the vascular cambium and includes the secondary phloem, secondary cortex and the periderm [1, 2]. Bark’s outer layer, or phellem, is composed of mostly dead tissues that form a protective barrier between plant and the abiotic and biotic environment, while the inner layer, or phloem, serves as a conduit for transport and storage of photosynthate [1, 2]. Despite its important roles including photosynthate transport [3], photosynthesis [4, 5], storage [6], mechanical support [7] and protection [8–11], the molecular basis of bark formation remains poorly understood (for reviews see: [12, 13]).

Bark texture varies among species, and even among genotypes within species, and has notable phenotypic diversity ranging from smooth, peeling, fractured, fissured to plated [14]. Within genera, bark texture differs between related species, e.g., in mature *Populus trichocarpa* (Torr. & Gray), bark is smooth or lightly flaky, while in *P. deltoides* (Bartr. ex Marsh), bark is rough and highly furrowed [15]. Bark’s high morphological diversity suggests that variation in texture may be an important component of variation in plant ecological strategies. It has been reported that in ash and beech smooth bark genotypes are less susceptible to insect and fungal diseases [16, 17]. In addition, bark thickness and moisture content are correlated with enhanced fire resistance [9, 18, 19] and in cork oak the phellem is also the basis of the cork manufacturing industry [20]. Despite the biological, ecological and industrial value of bark, the genetic basis of bark’s features remains undefined.

To further understand the variation in bark texture, we need a better understanding of outer bark development. The outer bark includes all tissues formed by the phellogen, consisting of dead hollow cork cells [1, 2], and originating from the outermost layer of the secondary phloem [2]. Romero [21] has proposed that discontinuous periderms may be the result of variation in radial meristematic activity in the phellogen in apparent response to the mechanical stresses imposed by radial growth, whereas, smooth textured barks may be derived from the formation of a single periderm and continuous shedding of phellem. However, since most plant species develop several periderms over the course of time, smooth bark scales can develop from preceding periderms from beneath the stem surface while uneven thick and thin layers result in the bark splitting, and in a peeling bark appearance [21]. Finally, Romero [21] also suggests that scaly and fissured bark develops when bark growth is discontinued and overlapping layers of periderms are formed. While these descriptions provide indications on how bark texture can vary, there remains a lack of understanding of phellem development at the molecular level. Additionally, variation in radial meristematic activity in the cambium may affect both diameter growth and bark texture as result of mechanical stresses. Especially Lateral Organ Boundaries (LBD) genes were found to be important regulators of woody perennial growth in poplar [22]. Specifically, the two LBD genes, PtaLBD1 and PtaLBD4 are expressed at the cambium/phloem boundary and are involved in ray cell and secondary phloem development. Two other LBD genes, PtaLBD15 and PtaLBD18, are expressed at the cambium/xylem boundary and are involved in secondary xylem development.

Quantitative Trait Locus (QTL) mapping in segregating populations is a powerful tool to 1) uncover genes underlying naturally occurring phenotypic variation and 2) dissect the genetic basis of phenotypic traits [23]. QTL-based approaches have often been implemented to study the complex genetic architecture underlying wood formation, including lignin, diameter, height, biomass and various wood chemistry traits [24–31]. However, only a few studies have explored bark features and are limited to bark thickness in *Eucalyptus globulus* [32], *Pinus* hybrids [33] and *Boehmeria nivea* [34]. In *Populus*, QTL mapping and gene expression analyses were used to link sequence polymorphisms and variation in transcript levels [35–37].

Thus, in this study, we investigate bark texture, bark thickness and diameter variation in the pseudo-backcross Family 52–124 derived from a cross between a *P. trichocarpa* x *P. deltoides* hybrid and *P. deltoides* [25, 38]. Novaes et al. [25] performed a QTL mapping study in the same mapping pedigree for 20 biomass and wood chemistry traits (including stem diameter) under different nitrogen treatments and identified a total of 63 QTL distributed across 14 chromosomes. In the present study, QTL mapping was done using a genetic map with high marker density anchored to the *P. trichocarpa* whole-genome assembly [39], which allowed us to determine the map position of QTL and identify underlying candidate genes. Specifically, we performed QTL analyses for bark texture, bark thickness and stem diameter and report results across three years and two geographic locations. These three traits have been chosen since we suspect an inter-connection between their development. Specific objectives were to: 1) identify positional candidate genes that underlie QTL for bark texture, bark thickness and diameter growth and 2) test reproducibility and consistency of QTL across years and environments.

## Methods

### Mapping population

An interspecific hybrid poplar pseudo-backcross pedigree (Family 52–124) composed of 396 genotypes was generated by crossing the hybrid female clone 52–225 (TD), an F_1_ hybrid derived from *P. trichocarpa* (TT, clone 93–968) × *P. deltoides* (DD, clone ILL-101), with *P. deltoides* (DD, clone D124) (Additional file 1: Figure S1a) [25]. The F_1_ hybrid, clone 52–225, had smooth bark and was crossed with *P. deltoides* clone D124 with rough bark. The mapping population was planted at Boardman, OR (45°50′8″N, 119°33′48″W) in 2010 on land owned by GreenWood Resources Inc. Two clones of 396 genotypes were planted in a three-block replication for a total of six ramets per clone (Additional file 1: Figure. S1b). The same pedigree was planted in Morgantown, WV (39°39′32″N 79°54′19″W) on the West Virginia University agriculture experimental farm in 2006 with four replicates of the 396 genotypes used in this study. Permission to collect data from both sites was covered under the collaborative efforts established in the BioEnergy Science Center initiative.

### Plant material and construction of genetic linkage map

We used the genetic map of the mapping family 52–124 comprised of 3568 SNP markers with known genomic positions for QTL identification. SNP genotyping, marker curation and genetic map construction were previously described by Muchero et al. [31].

### Phenotypic measurements

**Fig. 1:**
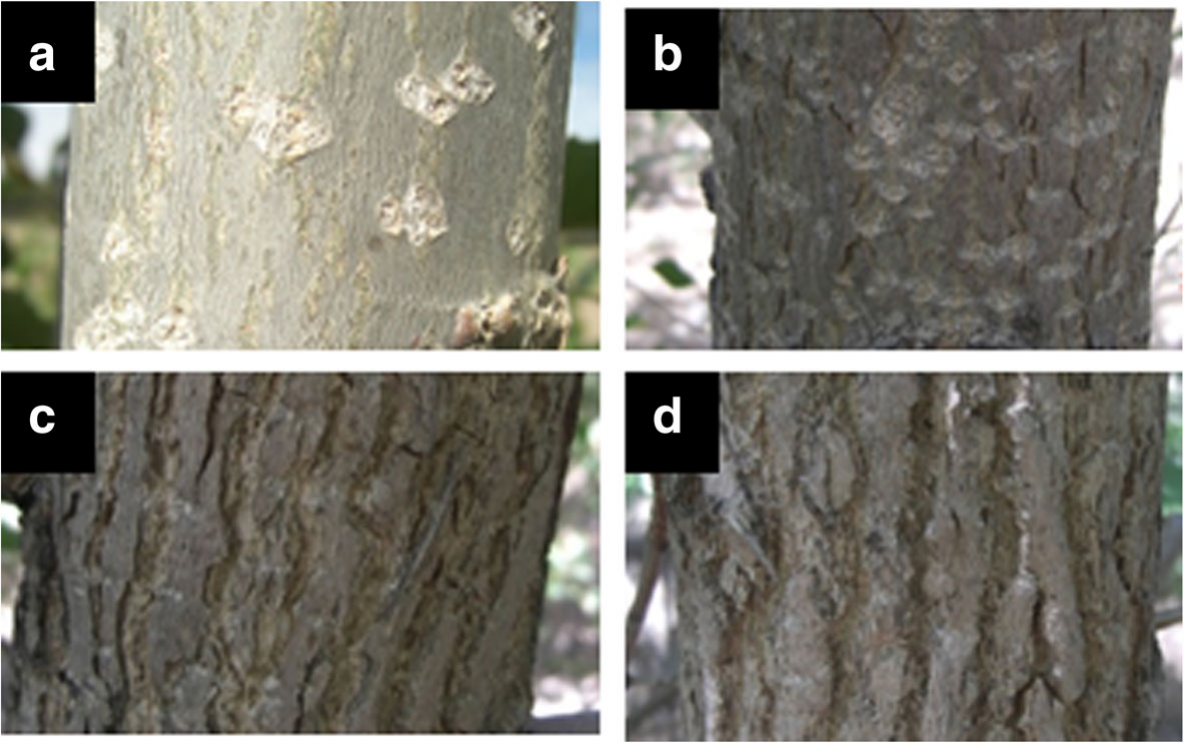
Bark texture scale for *Populus* Family 52–124 offspring. Ranging from smooth (**a**), medium (**b**), rough (c) and rough/deeply furrowed bark (**d**), and level 4 was only found in Oregon

Phenotypic data for bark texture (BT), bark thickness (BTh) and diameter at breast height (D) for all 396 full-sibs were analyzed in this study. Specifically, for the Oregon site (OR), bark texture data were collected in year 3, 4 and 5 by visual inspection, whereas diameter and bark thickness data were collected only in year 3 by using diameter tape and a bark thickness gauge on two opposites sides of the stem. For the West Virginia site (WV), bark texture data were collected in year 4 and 6 by visual inspection; diameter and bark thickness traits were collected only for year 4 using a caliper and ruler. Bark texture was assigned a qualitative score based on a scale from 1 (smooth) to 4 (furrowed with deep grooves) (Fig. 1). Especially, the replicated multi-year measurements allowed for a reliable identification of QTL in Boardman, Oregon. Some QTL in Morgantown, Virginia, could have remained undetected as result of the lower number of replicates and years.

Pair-wise phenotypic correlations were calculated as Pearson correlation coefficients for all three traits across the two different geographic regions and the three years using WinSTAT software [40] to asses covariance within blocks, and within and between years (Additional file 2: Table S1). To assess variation within blocks, the two replicates for each of three blocks, named as 1a, 1b, 2a, 2b, 3a & 3b, respectively, were analyzed at the OR site in year 3. In year 4 and 5, only block 1 and 2 were measured.

### QTL analysis

The data were evaluated for the presence of outliers and recording errors were corrected or deleted. WinSTAT [40] was used to check for normal distribution of residuals. Transformations were deemed unnecessary (Additional file 3: Figure S2). The traits were analyzed with the interval mapping method implemented in MapQTL6 [41] to map putative and suggestive QTL intervals on the genetic linkage map and to test for reproducibility across years and environments. Composite interval mapping with the Multiple-QTL Model (MQM) method was then used to further refine the QTL regions. Markers closely linked to a putative QTL were selected as cofactors and the selected markers were used as genetic background controls in subsequent MQM mapping. We selected additional cofactors until no additional QTL was detected. Mean phenotypic values across the two replicates per site were analyzed separately for each of the three blocks across three years for the OR site and across two years for the WV site. The putative QTL were subjected to 1000 genome-wide (GW) and chromosome-wide (CW) permutation tests [42] to determine LOD significance thresholds at the 0.05 significance level (Additional file 4: Table S2). A putative QTL was declared when it was detected in at least two replicates or in one replicate in different years or sites, with at least one of those instances exceeding the chromosome-wide LOD threshold. To account for minor deviations from normality in some cases, we also performed the non-parametric Kruskal-Wallis test which is the equivalent to the one-way analysis of variance [43].

### Candidate genes

Genes underlying genome-anchored QTL intervals (Additional file 5: Table S3 and Additional file 6: Table S4) were identified from the *Populus* genome assembly V3.0 [44] in the Phytozome database via BioMart tool (https://phytozome.jgi.doe.gov). A complete gene list with InterPro descriptions was collected including both putative and unknown functions. Expression profiles of the gene models from various tissues: bud, leaf, various parts of root and stem (expression FPKM), were downloaded from the publicly available PhytoMine database (https://phytozome.jgi.doe.gov/phytomine/begin.do). The Affymetrix microarray expression raw date profiles for the traits bark and mature phloem, developing phloem, cambium, developing cambium and mature cambium were obtained from the NCBI, GEO database (https://www.ncbi.nlm.nih.gov/geo/, GEO accession number GSE30507) [45]. The raw data were normalized using the RMA algorithm [46] and further analyzed statistically using TM4:MeV software [47, 48], utilizing Affymetrix probe annotation [49].

The genes’ expression in developing and mature phloem/xylem and cambium was then assessed for each QTL interval for all traits based on publicly available data. For each QTL cluster, genes in the map interval with the highest LOD score and high expression in phloem, cambium and xylem tissues (above the 90th percentile) were compiled in a list (Additional file 7: Table S5).

### Position of LBD genes

The position of the Lateral Organ Boundaries Domain (LBD) genes with putative role in bark development and diameter growth were identified by using the BLAST tool in the *Populus* genome assembly V3.0 in the Phytozome (https://phytozome.jgi.doe.gov/) database against well-established *Arabidopsis thaliana* LBD genes.

## Results

### Analysis of phenotypic correlations among traits and trait frequency distributions

Bark phenotypes ranged from smooth (1) to deeply furrowed bark (4) (Fig. 1). Shallowly fissured bark typical for *P. trichocarpa* was not found in this backcross pedigree (*P. deltoides* x *P. trichocarpa* hybrid backcrossed with *P. deltoides*). The interspecific crossing parent, *P. trichocarpa* (clone 93–968) x *P. deltoides* (clone ILL-101), had relatively smooth bark (mean value: 1.33, SD: 0.47). The other crossing parent, *P. deltoides* clone D124, had a rough bark texture (mean: 2.66, SD: 0.47). The grandparent *P. deltoides* (DD, clone ILL-101) had a slightly furrowed bark texture while grandparent *P. trichocarpa* (TT, clone 93–968) had smooth and slightly fissured bark (field observations on adult trees, no genotypes and measurements available in the field trials). Bark texture showed the highest correlation within blocks (*r* = 0.91 to 0.93, *p* ≤ 0.0001). The phenotypic correlations at the OR site within the same year for bark texture ranged from *r* = 0.58 to 0.76; for diameter, *r* = 0.38 to 0.45 and for bark thickness, *r* = 0.40 to 0.56, all at *p* ≤ 0.0001 (Additional file 2: Table S1). Comparing mean values of traits among years, bark texture values were significantly correlated among years at the OR site (*r* = 0.51 to 0.77, p ≤ 0.0001). The correlations were weaker for the WV site, but still highly significant (*r* = 0.39, *p* ≤ 0.0001). Finally, at year 3, bark thickness showed a strong positive correlation with both bark texture (*r* = 0.32 to 0.69, mean value *r* = 0.49, *p* ≤ 0.0001) and diameter (*r* = 0.17 to 0.75, mean value *r* = 0.43, *p* ≤ 0.0001) within the OR site (Additional file 2: Table S1, blue and green sections), however, bark texture and diameter showed inconsistency in correlation values and significance ranging from *r* = 0.15 (*p* ≤ 0.05) to *r* = 0.47 (*p* ≤ 0.0001) (Additional file 2: Table S1, red section). Overall, traits showed high correlations among replicates and years, and traits were correlated with each other. Across site correlations were only significant for bark texture ranging from *r* = 0.25 to 0.40 (*p* ≤ 0.01–0.0001).

### QTL analysis and detection across contrasting environments

**Fig. 2:**
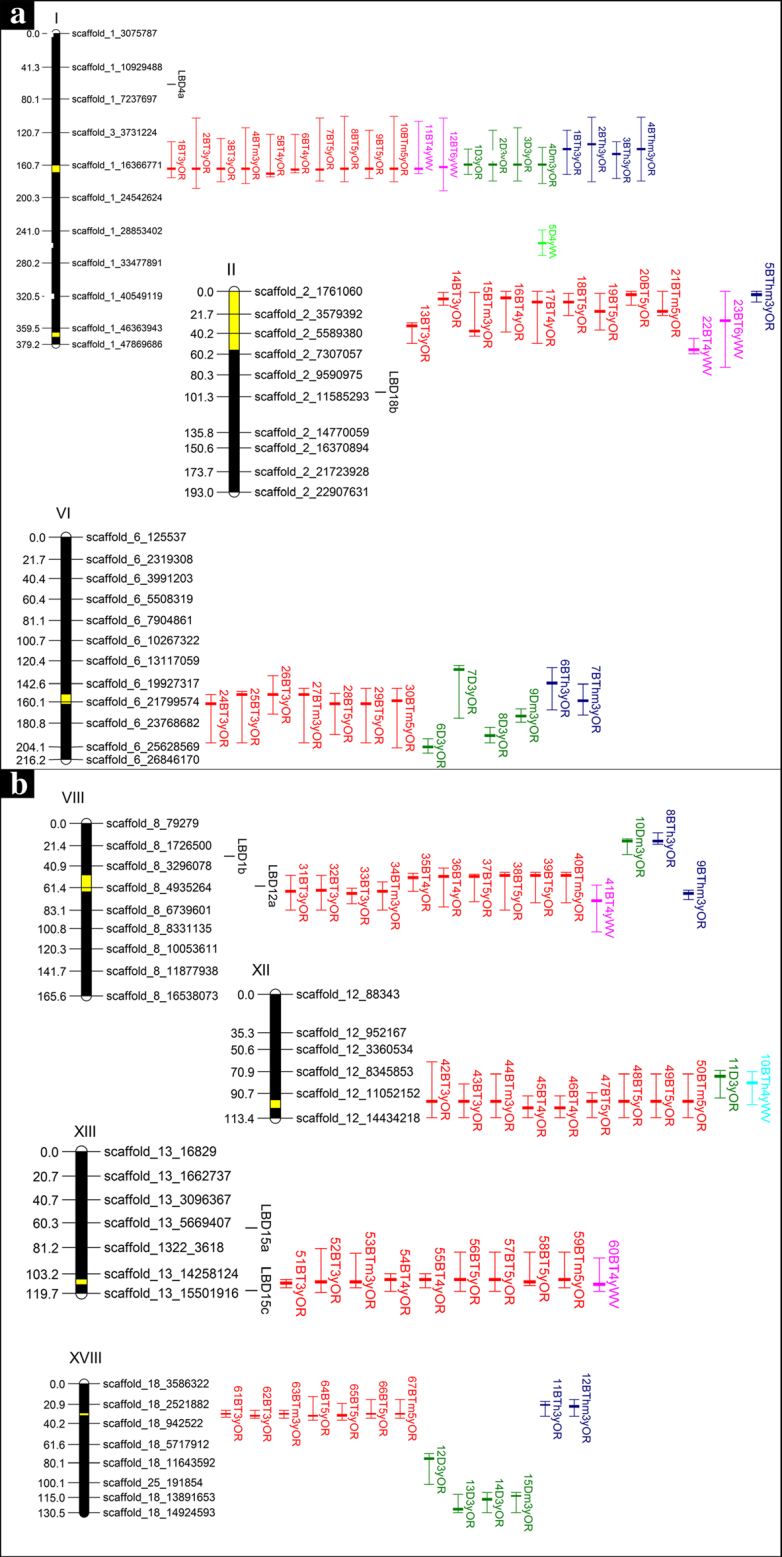
QTL anchored to the genome of *Populus trichocarpa* (V3 assembly, Kelleher et al., 2007). **a**: linkage groups I, II, VI, **b**: linkage groups XII, XIII, XVII. The actual map has a high marker density (average marker spacing: 5 markers per 4 cM). For illustration purposes, for each linkage group an evenly spaced selection of scaffolds is shown (1 marker per 20 cM). The yellow regions on LGs represent LOD score maxima across years and environments. QTL for bark texture (BT), diameter (D), and bark thickness (BTh) are shown in red, green, and blue for Oregon and in pink, light green, and turquois for West Virginia and named according to Additional file 4: Table S2. LOD score maxima, genome-wide intervals (solid bars) and chromosome-wide intervals are shown for QTL that were identified in different years and environments (see Additional file 4: Table S2). The outer lines of bars are CW thresholds and middle lines are QTL LOD maxima. The exact map and physical locations of QTL are shown in Additional file 4: Table S2. Scaffold intervals are represented in Mb. Black vertical lines represent the physical location of LBD genes in the *P. trichocarpa* genome, orthologues are notes by a, b or c

Seven major QTL clusters were detected for bark texture on seven individual chromosomes I, II, VI, VIII, XIII and XVIII (Additional file 4: Table S2), with all clusters containing at least three individual QTL above the GW threshold. For diameter, three QTL clusters with significance above the GW threshold were detected on chromosome I, VI and XVIII; in addition, two suggestive QTL above the CW threshold were detected on chromosome VIII and XII (Additional file 4: Table S2). Bark thickness showed three QTL clusters above the GW threshold on chromosome I, VI and XVIII, and four QTL above the CW threshold on chromosome II, VIII and XII (Additional file 4: Table S2). Chromosome VIII likely contains two separate QTL since they map to distinct chromosomal positions. All 94 individual QTL detected for the three traits across various chromosomes were successfully anchored to the *Populus* genome assembly (Fig. 2; Additional file 4: Table S2). For the seven bark texture QTL clusters, the percentage of phenotypic variance explained (PVE) ranged from 3.6 to 12.8% for QTL above the GW threshold, while for diameter and bark thickness, it ranged from 5.4 to 8.4% and 4.5 to 9.6%, respectively (Additional file 4: Table S2). For QTL on chromosome II, VI, VIII and XII the *deltoides* genotype DD was associated with the lower value for bark texture (Additional file 4: Table S2), while for QTL on chromosome I, XIII and XVIII the DT genotype was associated with a lower value for bark texture.

**Fig. 3:**
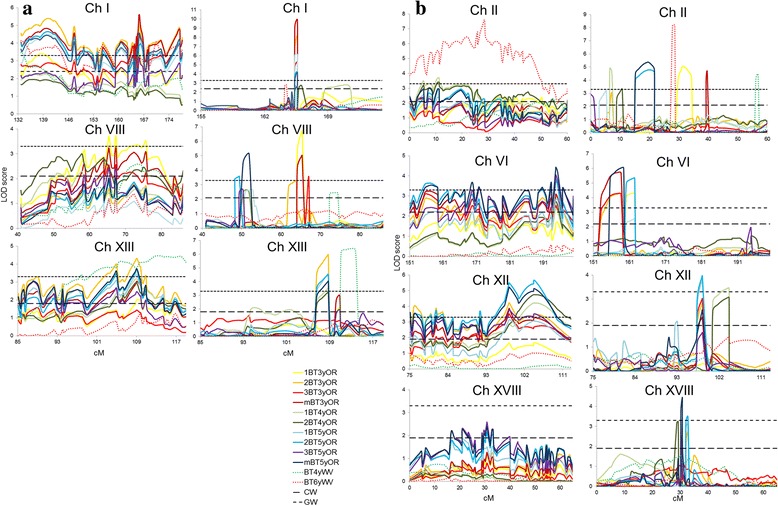
LOD score profiles for bark texture (BT) QTL detected in the *Populus* Family 52–124. Specifically, BT QTL detected on **a** LG I, VIII and XIII and **b** LG II, VI, XII and XVIII using interval mapping (left) and the Multiple-QTL Model (MQM) with co-factor selection (right) across all experimental replicates at Oregon (OR) across 3 years and at West Virginia (WV) across 2 years. Chromosome-wide (CW) and genome-wide (GW) significance thresholds are shown with dashed lines (α = 0.05, 1000 permutations). Yellow, orange, and red solid lines represent LOD score profiles for 3-year-old replicated samples at Oregon, solid shades of green lines for 4-year-old samples, and shades of blue lines for 5-year-old samples. Dotted red and green lines represent LOD profiles for 4-year-old and 6-year-old samples at the West Virginia site. Two broken horizontal lines represent GW and CW LOD significance threshold after 1000 permutations at the *p* ≤ 0.05 significance level. The exact map and physical locations of QTL are shown in Additional file 4: Table S2

Figure 3 shows a graphical outline of LOD score profiles for bark texture QTL versus map location across all seven chromosomes before (left) and after cofactor selection (right). The QTL for bark thickness and diameter, described above, overlap with six out of the seven bark texture QTL clusters using the interval mapping approach (Fig. 2). Specifically, QTL for bark texture overlap with diameter and bark thickness QTL on chromosome I, VI and XII, and solely with bark thickness QTL on chromosome II, VIII and XVII (Fig. 2). Overall, reproducibility and co-location within the same map interval are observed across experimental replicates within sites and years and in some cases across sites.

### Description of QTL clusters

Based on significance, consistency and reproducibility of the QTL across sites, years and replicates, specifically for bark texture, we classified the QTL clusters according to four criteria: 1) significance (LOD scores), 2) reproducibility across biological replicates, 3) reproducibility over time (years) and 4) reproducibility across environments (sites). All seven QTL clusters were significant with at least three individual QTL having LOD scores above the GW threshold and were reproducible across blocks (biological replicates) within the same year and across two years for the OR site. Four QTL clusters on chromosome I, II, VIII and XIII, were reproducible between sites across very different environments. In each QTL cluster most of the QTL were associated with bark texture. Below we provide a detailed description of all seven bark texture QTL clusters ranked according to the four criteria and their association with bark texture, bark thickness and diameter.

QTL cluster on chromosome I was associated with all three traits (Fig. 2). For diameter four out the five QTL were significant at the GW threshold and for bark thickness three out of the four QTL were above the GW threshold (Additional file 4: Table S2). For bark texture, twelve QTL were detected across all replicates and years in both OR and WV, and ranged from 101 to 192 cM (before cofactor selection) with highly reproducible LOD maxima after cofactor selection consistently around 165 cM (Additional file 4: Table S2; Fig. 3a). For the OR site, seven out of the ten QTL in this cluster were above the GW threshold all being detected in years 3 and 5; whereas at the WV site, the two QTL for both years were above the CW threshold. All twelve individual QTL mapped reproducibly to the same map interval and LOD maxima positions at the GW threshold were typically associated with no more than three markers in close proximity, around 3 cM (Fig. 3a). Notably, the results showed consistency and high reproducibility, first between blocks within individual years, second within the same site, and third across sites. Overlapping QTL intervals and maxima with high significance across all years are presented in Additional file 4: Table S2 and in Figs. 2, 3.

The second bark texture QTL cluster was detected across all replicates and years in OR, and in year 4 in WV, and was mapped on chromosome VIII within the chromosomal region 48 to 104 cM before cofactor selection with varied LOD maxima positions after cofactor selection (~50 cM, ~65 cM or 74 cM) (Additional file 4: Table S2; Fig. 3a). When comparing this cluster with other traits, one overlapping QTL for bark thickness (significant at the CW level), peaking at 67 cM was detected. For the OR site, six of the ten QTL for bark texture in this cluster were significant above the GW threshold; whereas at the WV site, only one QTL was found above the CW threshold. All eleven individual QTL within this cluster mapped reproducibly within the same map interval, however positions of QTL maxima varied for the OR site (Fig. 3a). The overall reproducibility within the OR site, across both sites and across all years, was high and QTL were found within the same chromosomal region covering 25 cM.

The third bark texture QTL cluster mapped on chromosome XIII and was detected across all years and replicates in OR, and in year 4 in WV, within the chromosomal region 83 to 118 cM before cofactor selection and reproducible LOD maxima after cofactor selection were found around 108 cM (Additional file 4: Table S2; Fig. 3a), which interestingly co-located with the Lateral Organ Boundaries Domain gene LBD15c (Potri.013G156200), a candidate gene for xylem development [22]. No QTL for bark thickness or diameter were found in this QTL interval. For the OR site, seven out of the nine QTL in this cluster were significant above the GW threshold across all years; whereas at the WV site, only one QTL was detected and found above the GW threshold. All ten individual QTL mapped reproducibly within the same map interval and positions of QTL maxima were typically associated with no more than four markers in close proximity covering a map interval around 5 cM (Fig. 3a). The results showed overall consistency and reproducibility between block replicates within individual years, within the OR site and across the two sites and across most years (Additional file 4: Table S2, Fig. 3a).

The fourth bark texture QTL cluster on chromosome II was detected in both OR and WV, across all years and replicates for both sites, within a chromosomal interval from 0 to 70 cM (before cofactor selection), but displayed inconsistent and variable LOD maxima after cofactor selection (Additional file 4: Table S2; Fig. 3b). When comparing this cluster with bark thickness and diameter, one overlapping QTL for bark thickness (significant at the CW threshold), peaking at 0 cM, was found. Eight out of the ten individual bark texture QTL were significant above the GW threshold, indicating reproducibility within replicates and sites, between sites and across years. However, after cofactor selection, the LOD maxima greatly varied even within replicates of the same year.

The fifth bark texture QTL cluster was detected on chromosome VI in OR in years 3 and 5 only, within a chromosomal interval from 135 to 204 cM (before cofactor selection) and had LOD maxima after cofactor selection between 153 and 162 cM (Additional file 4: Table S2; Fig. 3b). Several overlapping QTL, four for diameter (two GW QTL) and two GW QTL for bark thickness, were found within the same chromosomal interval, however the LOD maxima varied among traits after cofactor selection. Six out of the seven individual QTL for bark texture were above the GW threshold, indicating reproducibility within replicates at the OR site and across the two years. However, the results were not consistent across the two sites, as no QTL was detected at the WV site. Only three out of the nine individual QTL were found significant above the GW threshold, while others were significant at the CW threshold.

The sixth bark texture QTL cluster mapped on chromosome XII and was detected across all years and replicates in OR within the chromosomal interval from 62 to 113 cM before cofactor selection and consistent LOD maxima between 98 and 104 cM were detected after cofactor selection at the OR site (Additional file 4: Table S2; Fig. 3b). Two individual QTL, one for bark thickness (CW QTL) and one for diameter (CW), were partially overlapping with bark texture QTL, but had LOD maxima separate from the LOD maxima for bark texture. Out of the nine individual QTL, only three were above the GW threshold. The results are reproducible between replicates and within and across the three years in OR. Though no significant QTL were detected in WV, the QTL graph shows a suggestive QTL with increasing LOD score near the same chromosomal interval noted above (Fig. 3b), though still below the CW threshold.

Finally, the seventh bark texture QTL cluster, mapped on chromosome XVIII within the chromosomal interval from 13 to 35 cM, before cofactor selection, with reproducible LOD maxima at 30 cM after cofactor selection, detected in year 3 and 5 at the OR site (Additional file 4: Table S2 and Fig. 3b). A QTL cluster for bark thickness closely overlapped with the same chromosomal interval with LOD maxima further upstream. Four separate QTL for diameter were detected on the same chromosome (two above the GW threshold). In year 5, three out of the seven bark texture QTL were above the GW threshold. The QTL were only reproducible across year 3 and 5 at the OR site, although a suggestive QTL was found for WV (Fig. 3b).

The Kruskal-Wallis rank sum test was subsequently used to confirm significant associations of individual markers linked to the QTL. For all traits, the markers underlying the QTL interval were also significantly associated with the traits (*p* ≤ 0.005). The Kruskal-Wallis test provides further confirmation of the marker-trait association, indicating that the results of the QTL analysis were not influenced by segregation distortion or non-normal distribution of certain traits.

### Candidate gene identification and characterization

To narrow the QTL position and identify candidate genes within the QTL interval MQM mapping was used. Intervals spanning the genomic regions (physical location by MQM mapping) summarized in Additional file 4: Table S2 were used to identify all genes occurring within the seven QTL clusters for bark texture and for the QTL clusters that were associated with bark texture, diameter and bark thickness. The number of genes for each trait in QTL clusters based on MQM mapping with cofactor selection is summarized in Additional file 5: Table S3. There were 1869 genes within genome-anchored QTL intervals for bark texture, out of which, 1476 (82%) had annotations based on the InterPro domain and expression profiles (average FPKM) for 22 different tissues and based on Affymetrix microarray expression data for bark and woody tissues (phloem, cambium and xylem), whereas a total of 693 and 789 genes were detected in QTL clusters for diameter and bark thickness, respectively (Additional file 6: Table S4).

QTL for all three traits overlap in QTL cluster on chromosome I, where the diameter QTL included 25 genes and the bark thickness QTL encompassed 115 genes. Two additional QTL clusters on chromosome VI and XII were associated with all three traits where diameter and bark thickness QTL had 369 and 209 overlapping genes on chromosome VI and 29 and 14 overlapping genes on chromosome XII, respectively. QTL clusters for bark texture on chromosome II, VIII and XVIII overlapped with bark thickness QTL only, containing 963, 23 and 38 overlapping genes, respectively (Fig. 2, Additional file 6: Table S4). As a result of the Salicoid duplication event in the *Populus* genome, nearly every chromosome has a paralogous segment elsewhere in the genome; this is due to the whole-genome duplication between chromosomes resulting in homologous genomic blocks [50]. Each of the seven chromosomes noted above has a Salicoid paralog, yet none of these duplicated genes in paralogous segments co-located with other QTL for the same trait.

Top expressional candidate genes (above the 90th percentile) for mature and developing phloem, cambium, developing xylem and mature xylem in QTL intervals with the highest LOD scores resulted in a compiled list of the top candidate genes for each trait (Additional file 5: Table S5). In total, the top candidate genes with putative function in the control of bark texture, diameter and bark thickness are narrowed down to 40, 20 and 46 genes spanning various QTL clusters (Additional file 7: Table S5).

## Discussion

We have characterized segregating bark features in an interspecific backcross of *Populus*. With the use of QTL mapping, we are able to link the phenotypic traits to their associated polymorphisms in the genome, thus integrating phenotypic and genotypic data to identify putative genetic mechanisms related to phellem development.

While other studies have identified QTL in interspecific *P. trichocarpa* and *P. deltoides* families for many different traits, including leaf size and shape, growth and bud set, diameter, height, stem and root biomass and various wood chemistry phenotypes [25, 35, 51–54], little research has been done on bark features [55–57], despite bark being one of the key-energy-related characteristics of lignocellulosic feedstock [58–60].

We identified several QTL that encompassed both bark traits and stem diameter and found that these intervals mapped consistently across geographic locations, replicates within sites and across years. Interestingly, one study, using the same pedigree, reported several overlapping QTL with our traits [25]. Specifically, bark texture QTL in our study overlapped on chromosome I, II and XVIII with QTL associated with total biomass, C5 and C6 sugars, and height. Additionally, diameter and bark thickness QTL overlapped on chromosome VIII, XII and XVIII with QTL for diameter and biomass traits [25]. It is difficult to determine if there are genes that have pleiotropic effects or whether there are alternate genes within the co-located intervals because of the large size of the interval and lack of expression evidence in the Novaes et al. [25] study.

While a few studies have analyzed bark thickness, e.g., *Eucalyptus globulus, Pinus* hybrids and *Boehmeria nivea* [32–34], the genetic basis and causal loci of bark thickness and/or bark texture have not yet been determined. In *Boehmeria nivea,* a perennial herbaceous plant belonging to the Urticaceae family, several QTL for bark thickness have been mapped and some were identified in the same QTL intervals across two contrasting environments in Changsha, China at varying time throughout the year [34].

Bdeir et al. (2016) and Yordanov et al. (2010) previously identified genes with a role in bark development [22, 61]. Based on the generation of loss-of-function phenotypes through transgenic plants, Lateral Organ Boundaries Domain (LBD) genes were found to have a crucial role in meristem maintenance and were identified as important regulators of woody perennial growth in poplar, specifically in *Populus tremula* x *Populus alba* (Pta). The overexpression of PtaLBD1 resulted in wide multiseriate rays as compared to uniseriate rays in the wild type [22]. In the regulation of secondary (woody) growth, two genes of the LBD Family (PtaLBD1 and PtaLBD4) were involved in secondary phloem and ray cell development and two genes (PtaLBD15 and PtaLBD18) in secondary xylem formation. Interestingly, one of the PtLBD15 paralogs (Potri.013G156200, total expression 36.30), previously found to have a role in secondary growth, was found in the QTL cluster on chromosome XIII for bark texture with the highest LOD score. In this chromosome region seven out of a total of 10 QTL detected were above the genome-wide threshold (Fig. 2). PtaLBD1 (Potri.008G043900) was detected in the diameter QTL cluster on chromosome VIII with a LOD score of 2.27. Both PtaLBD15 and PtaLBD1 are involved in secondary growth in poplar and are potential candidate genes for diameter growth and bark characteristics. PtaLBD15 was found in *Populus tremula* x *P. alba* to be mainly expressed at the cambium/xylem boundary and thus is likely involved in secondary xylem development. PtaLBD1 was found to regulate secondary phloem and ray development and was highly expressed in the phloem and cambial zone [22]. Therefore, their apparent involvement in secondary growth and development in poplar and their detection in QTL clusters for diameter and bark texture make them candidate genes for these traits. Finally, PtaLBD12 (Potri.008G072800, total expression 26.06) was detected in the QTL cluster on chromosome VIII and overlapped with diameter and bark thickness QTL. PtaLBD12 has been reported to be involved in the development of various lateral organs from the meristem in *Arabidopsis* plants, but its role in secondary growth is unknown [62].

We were able to identify a list of candidate genes underlying the QTL intervals of all three traits using genetic markers anchored to the *Populus trichocarpa* genome. Generally, QTL were highly reproducible among biological replicates and years and even across geographic locations. While some QTL studies obtained good reproducibility across two different time data set [33, 34, 63], our study has identified significant QTL consistently co-locating across sites, years and replicates especially for bark texture. For instance, QTL in clusters on chromosome I, VIII, XII, XIII and XVIII were consistently identified within ~25 cM.

When comparing all the aspects of the seven bark texture QTL clusters, including significance, reproducibility across replicates, years and sites, along with consistency of the QTL, major QTL were identified on chromosome I, VIII and XIII, where QTL maxima were found within a 5–20 cM interval across most replicates, years and at both sites. Given the environmental contrast between the OR and WV experimental sites, four out of the seven QTL clusters, representing a total of 47 individual QTL, detected for bark texture were remarkably consistent across both sites. Differences in reproducibility for QTL clusters across sites suggest differential environmental effects on gene expression. In comparison to bark texture, QTL clusters for bark thickness and diameter had lower reproducibility across sites.

In QTL clusters on chromosome I, VI and XII, QTL for bark texture, bark thickness and diameter were syntenic. Co-location of QTL for traits can be the result of pleiotropic effects or closely linked genes. These overlapping QTL could be an explanation of different aspects of bark texture and radial growth. Romero [21] proposed that rough bark results in response to the mechanical stresses imposed by a varied radial growth and due to different meristematic activity in the phellogen, a discontinuous periderm. Strong correlation between bark texture and diameter could indicate that bark texture is partly related to diameter growth. Using interval mapping without cofactor selection, QTL for bark texture overlap with QTL for both traits which are related to radial growth (diameter and bark thickness) on three chromosomes (I, VI, XII) and solely with bark thickness on three other chromosomes (II, VIII, XVII). Furthermore, using MQM mapping, QTL for these traits were mapped to different neighboring positions of the same chromosomes (Fig. 2). Consequently, bark texture seems to be only partly related to diameter growth, and other factors such as meristematic activity of the phellogen and cell adhesion are likely to have major effects on bark texture too. A higher mapping resolution as obtained in linkage disequilibrium mapping in natural population samples is needed to narrow down the QTL region to individual genes and to distinguish between pleiotropic effects and close linkage.

Bark features in our study ranged from smooth to deeply furrowed which is characteristic for *P. deltoides*. Variation in shallowly fissured bark which is characteristic for *P. trichocarpa* was not observed among the segregating progeny. Thus, the QTL identified in this progeny set only represent a subset of a larger number of polymorphisms affecting the traits. And in a pseudo-backcross involving multiple *P. deltoides* parents, polymorphisms associated with characteristic bark features of *P. trichocarpa* seem to be largely undetected. Association populations for *P. trichocarpa* will be used to find additional candidate genes associated with bark texture in this species.

Each of the seven QTL clusters detected has a Salicoid paralog, and yet none of these paralogous genes showed up in the QTL analyses as significant. This further supports that the identified chromosomal regions are not artifacts of spurious correlations. Due to large genomic intervals in QTL clusters with partly overlapping QTL intervals the identification of specific candidate genes was difficult. This limitation was evident in our analyses where only two out of the seven QTL clusters on chromosome XIII and XVIII, encompassed less than 70 candidate genes, while the other clusters included from 123 to 963 candidate genes. Nonetheless, several candidate genes within the QTL interval can be identified based on their putative functions. Using the MQM method, we were able to identify informative loci for bark texture and narrow the QTL region to a small chromosomal region with a short and manageable candidate gene list. For example, a total of 11 NAC genes were detected in bark texture QTL clusters, one, five, one, three and one paralogs found on chromosome I, II, VI, VIII and XII, respectively. Specifically, the gene PopNAC128 (Potri.001G206900) is a prime candidate gene and was identified in the QTL interval on LG I within the QTL maxima (LOD score 9.88) and with a moderate expression value. PopNAC128 is one of the orthologs of *Arabidopsis* ANAC104 (*Arabidopsis* Nac Domain Containing Protein 104) and XND1 (Xylem NAC Domain 1). In a related study, *Populus* and *Arabidopsis* transgenic plants with overexpression of these genes resulted in severe dwarfing, lacking phloem fibers and a reduction in stem diameter, cell size and number, vessel number, and frequency of rays in the xylem [64]. While this study did not focus on bark texture, the lack of sufficient lignified fiber cells in the mutant affects the development of fiber bundles and ultimately bark texture as result of slowed secondary phloem development.

Another interesting gene, Potri.001G206700, an ortholog of AT4G33430 (BAK1, Bri1-Associated Receptor Kinase; ELONGATED; SERK3), is involved in patterning and growth regulation [65–67] and was found in the QTL interval on chromosome I, also within the QTL maxima (LOD score 9.88) and with a very high expression value above the 90th expression percentile across phloem/xylem and cambium tissues.

Variation in bark texture could be related to cell adhesion, which is essential to form a single periderm resulting in smooth bark, while lack of cell-cell adhesion leads to the development of uneven and discontinued bark or bark splitting causes a peeling and fissured bark appearance [21]. At the molecular level, several QTL and expressional candidate genes with high expression in phloem/xylem and cambium identified in this study have a role in cell adhesion, including Pinin (Potri.001G208200) and PopFLA or Fasciclin-Like Arabinogalactan (Potri.013G151300, Potri.013G151400 and Potri.013G151500). Interestingly, both of these genes fall within the QTL interval with the highest LOD scores, and also are above the 90th gene expression percentile for both xylem and phloem tissues. Many of the studies on Pinin, mainly on animal epithelial cells, revealed a vital role in cell-cell adhesion and cell shape [68, 69]. No studies exploring the Pinin gene in plants were found. The FLA gene is better studied across the plant kingdom, including *Arabidopsis* and *Populus*, and shows specific and high expression during the onset of secondary-wall cellulose synthesis, particularly in stem cells undergoing secondary-wall deposition [70, 71]. Transgenic lines indicate a role in cell-wall architecture and composition. Specifically for PopFLA, a role in tension wood formation in the xylem of mature stems was suggested based on a reduction in transcript levels leading to reduced stem flexural strength by modulation of cellulose and lignin composition in the xylem [70, 72, 73].

The top QTL and expressional genes revealed additional potential candidates (Additional file 7: Table S5), some of which are proteins of unknown or putative function and have never been studied. These genes represent potential candidate genes for future studies using either functional genomics or forward genetics techniques. Candidate genes within QTL intervals were identified based on Affymetrix Microarray expression profiles obtained from public databases. In the future, qRT-PCR confirmation of candidate genes’ expression profiles should be performed in various tissues (phloem, cambium, xylem, phellogen) of the parental clones and part of the mapping pedigree.

## Conclusions

In conclusion, the results presented in this study provide a basis for future genomic characterization of genes found within the QTL for bark texture, bark thickness and diameter in order to better understand stem and bark development in *Populus* and other woody perennial plants. Additionally, profiling the expression of the candidate genes (eQTL studies) in the developing bark of the mapping pedigree would allow parsing the list of candidate genes into those genes with high expression profiles in the tissue of interest. Bark texture is a complex trait which can be affected by differences in cell adhesion and radial meristematic growth. In the future, developmental differences between bark texture phenotypes should be analyzed in anatomical sections in representative genotypes and developmental stages.

## Additional files

Additional file 1: Figure S1. Mapping pedigree and block layout for *Populus* Family 52–124 used in this study. a) Family 52–124 is an pseudo-backcross pedigree between clone 52–225, an F_1_ hybrid derived from *P. trichocarpa* (T), 93–968 X *P. deltoides* (D) (ILL-101), back-crossed to *P. deltoides* (clone D124 (Novaes et al. 2009). Shading in pedigree: black = TT, gray = TD, White = DD genotype. b) Progeny plantation replicates and block layout in Oregon. Two adjacent replicates in each block and each genotype are represented in three blocks (6 replicates in total). Only two genotypes out of 392 genotypes are shown as an example. (TIFF 37 kb)

Additional file 2: Table S1. Pair-wise estimates of phenotypic correlations calculated as Pearson correlation coefficients between bark texture (BT), diameter (D) and bark thickness (BTh) phenotypes collected from *Populus* Family 52–124. Note: Specifically, the analysis was done within and between experimental blocks, across years and across sites. Symbols show *p*-values, where *p* ≤ 0.05 = *, *p* ≤ 0.01 = **, *p* ≤ 0.001 = ***, and *p* ≤ 0.0001 = ****. White cells are correlations between the same traits across years and sites; red cells are correlations between diameter and bark texture; green cells are correlations between bark thickness and bark texture; blue cells are correlations between bark thickness and diameter. (XLSX 158 kb)

Additional file 3: FigureS2. Frequency distribution for bark texture, diameter and bark thickness (a, b, and c, respectively) across Oregon and West Virginia sites and various years in *Populus* Family 52–124. NOTE: All supporting tables, except for Table S3, are in excel format submitted as separate files. (TIFF 209 kb)

Additional file 4: Table S2. QTL associated with bark texture, diameter and bark thickness identified in *Populus* Family 52–124 in Oregon and West Virginia. Note: Chr: chromosome; V2: markers anchored on version 2 of the *P. trichocarpa* genome; V3: version 3 updated physical location; PVE: percent phenotypic variance explained; DD: homozygous for the *P. deltoides* allele, DT: heterozygous for the *P. deltoides* and *P. trichocarpa* alleles. LOD max determined using MQM mapping, value with *: above GW threshold, otherwise above CW threshold. Alternating white or grey shades represent unique QTL found in one or multiple replicates across years and sites. Indexes 1, 2, 3 or m designate: replicate one, two, three or replicates’ mean value for OR samples, whereas for WV samples only one replicate was available; BT: bark texture; D: diameter; BTh: bark thickness; 3y, 4y, 5y or 6y: # years old; OR: Oregon, WV: West Virginia. (Example for population: 1BT3yOR: replicate one, bark texture, 3 year old samples, Oregon site). (XLSX 19 kb)

Additional file 5: Table S3. Number of candidate genes detected across QTL for the three traits. Note: The number of genes for each trait in QTL clusters based on MQM mapping with cofactor selection, sorted by significance and reproducibility. (DOCX 13 kb)

Additional file 6: Table S4. All candidate genes within the ninety four QTL detected in *Populus* Family 52–124. Physical localization, annotation and expression profile of gene models within each QTL interval for all traits. (XLS 4640 kb)

Additional file 7: Table S5. The 90th percentile candidate genes within the ninety four QTL detected in *Populus* Family 52–124. Physical localization, annotation and expression profile of gene models in the 90th percentile with high expression within LOD peaks for each QTL interval for all traits. (XLSX 78 kb)

## Abbreviations

BT: Bark texture
BTh: Bark thickness
CW: Chromosome-wide permutation tests
D: Diameter at breast height
GW: Genome-wide permutation tests
LBD: Lateral Organ Boundaries Domain
OR: Oregon site
PVE: Phenotypic variance explained
QTL: Quantitative trait loci
WV: West Virginia site
