# Supplementary figures and images for: Quantitative trait locus mapping of Populus bark features and stem diameter
Source: BMC Plant Biol. 2017 Nov 28;17:224. doi: 10.1186/s12870-017-1166-4 (PMC5704590; doi:10.1186/s12870-017-1166-4)

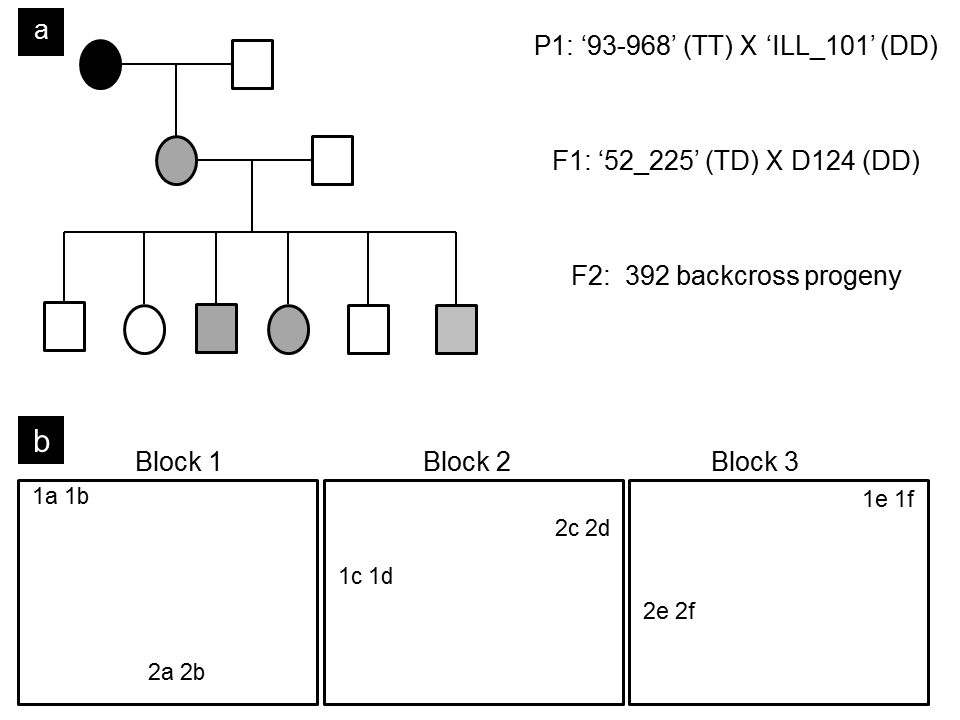

Supplement: Supplementary file 1 — Mapping pedigree and block layout for Populus Family 52–124 used in this study. a) Family 52–124 is an pseudo-backcross pedigree between clone 52–225, an F1 hybrid derived from P. trichocarpa (T), 93–968 X P. deltoides (D) (ILL-101), back-crossed to P. deltoides (clone D124 (Novaes et al. 2009). Shading in pedigree: black = TT, gray = TD, White = DD genotype. b) Progeny plantation replicates and block layout in Oregon. Two adjacent replicates in each block and each genotype are represented in three blocks (6 replicates in total). Only two genotypes out of 392 genotypes are shown as an example. (TIFF 37 kb) [file 12870_2017_1166_MOESM1_ESM.tif]

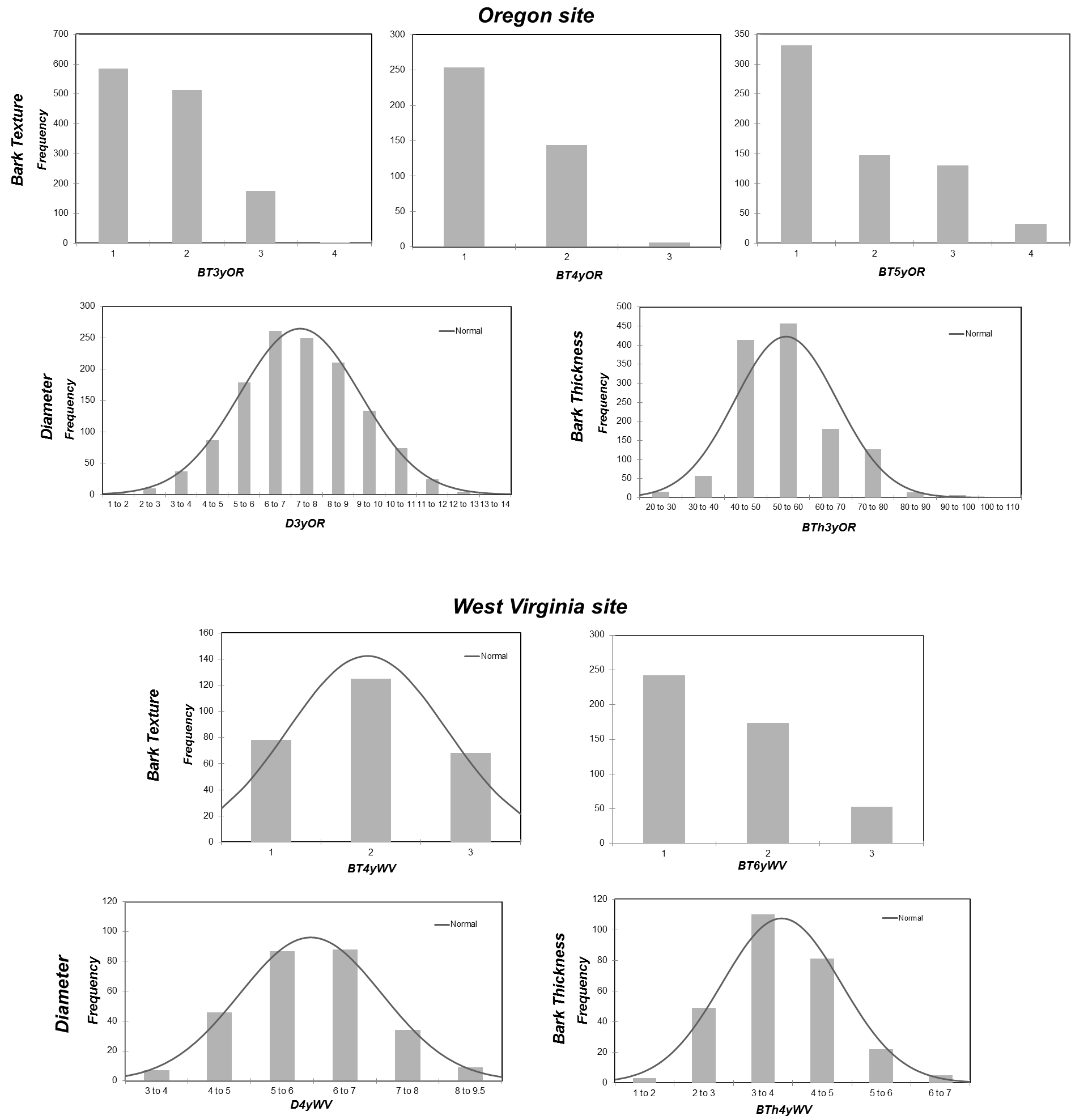

Supplement: Supplementary file 3 — Frequency distribution for bark texture, diameter and bark thickness (a, b, and c, respectively) across Oregon and West Virginia sites and various years in Populus Family 52–124. NOTE: All supporting tables, except for Table S3, are in excel format submitted as separate files. (TIFF 209 kb) [file 12870_2017_1166_MOESM3_ESM.tif]
