# Supplementary material for: Quantitative trait locus mapping of Populus bark features and stem diameter
Source: BMC Plant Biol. 2017 Nov 28;17:224. doi: 10.1186/s12870-017-1166-4 (PMC5704590; doi:10.1186/s12870-017-1166-4)
Supplement: Supplementary file 5 — Number of candidate genes detected across QTL for the three traits. Note: The number of genes for each trait in QTL clusters based on MQM mapping with cofactor selection, sorted by significance and reproducibility. (DOCX 13 kb) [file 12870_2017_1166_MOESM5_ESM.docx]

| **Table S3** Number of candidate genes detected across QTL for the three traits. | | |
| --- | --- | --- |
| Trait | QTL on Chromosome | Number of genes |
| Bark texture | I | 148 |
|  | VIII | 321 |
|  | XIII | 84 |
|  | II | 978 |
|  | VI | 130 |
|  | XII | 161 |
|  | XVIII | 47 |
| Diameter | I (~16820000bp) | 16 |
|  | I (~31760000bp) | 23 |
|  | VI | 362 |
|  | VIII | 45 |
|  | XII | 16 |
|  | XVIII | 231 |
| Bark thickness | I | 175 |
|  | II | 303 |
|  | VI | 208 |
|  | VIII (~157000bp) | 16 |
|  | VIII (~550000bp) | 19 |
|  | XII | 16 |
|  | XVIII | 52 |
| The number of genes for each trait in QTL clusters based on MQM mapping with cofactor selection, sorted by significance and reproducibility. | | |
